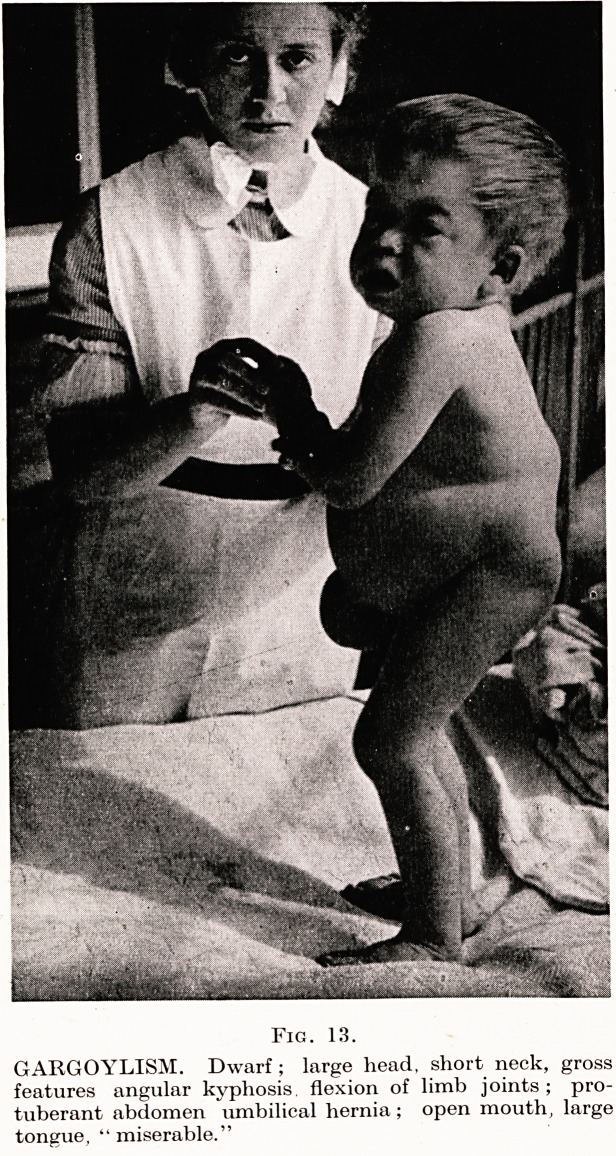# Unusual Children

**Published:** 1949-10

**Authors:** A. V. Neale, Beryl D. Corner, John Lowe

**Affiliations:** Professor of Child Health in the University of Bristol; Paediatrician, United Bristol Hospitals and Southmead Hospital; Medical Superintendent, St. Margaret's Hospital, Stratton St. Margaret, Wilts


### The Bristol Medico-Chirurgical Journal

*A Journal of the Medical Sciences for the*

### West of England and South Wales

*" Scire est nescire, nisi id me*
*Scire alius sciret."*

OCTOBER, 1949.

### UNUSUAL CHILDREN

BY

A. V. NEALE, BERYL D. CORNER AND JOHN LOWE.

A. V. NEALE, M.D., F.R.C.P.
*Professor of Child Health in the University of Bristol.*

### I : CONGENITAL SYPHILIS : WITH GANGRENE OF THE EXTREMITIES

CONGENITAL syphilis still occasionally escapes early diagnosis, which is unfortunate in view of the great value of modern remedies. This boy presented no outstanding diagnostic evidence at birth, but during subsequent months rhinitis and rash on the nates appeared and he generally failed to thrive. However, syphilis was not diagnosed until the boy was nearly two years of age, and by that time he was a miserable, listless boy with frontal bossing of the head and a marked rhinitis. A most unusual clinical development led up to the full investigation and diagnosis. Severe intermittent cyanotic congestion occurred in the hands and feet. The right hand was cyanosed and œdematous with marked dark discoloration of the fingers. On the left hand similar changes were seen in the distal phalanges of the third, fourth and fifth fingers. Foci of gangrene also appeared on several toes. The case shows the favourable progress following intensive therapy. Peripheral vascular effects may appear in congenital syphilis and it is very urgent that these should be recognized for (*a*) diagnostic significance, (*b*) intensive treatment.

In the present case a few weeks' delay probably determined the necrosis in the right hand.

CASE HISTORY.—A. B., boy aged two years, was admitted to County Hospital on February 18th, 1948, with discoloration of extremities of one week's duration and rapid onset.

*Previous History.*—Full term infant, normal delivery. No stigmata at birth ; later developed rash on nates and rhinitis which persisted ; no acute illnesses, but failed to thrive. Second child in family. Brother aged seven years, healthy, blood W.R. negative. Third child, born five-weeks-premature, died at one month with alleged broncho-pneumonia. Parents denied history of syphilitic lesions, but Wassermann tests were positive.

*On Examination.*—Miserable, listless ; marked rhinitis and frontal bossing of head ; skin dry, hair sparse ; rash on face and buttocks ; no abnormality of viscera detected ; C.N.S. normal.

Extremities : right hand cyanosed, œdematous with fingers discoloured and tender ; left hand, distal phalanges of third, fourth and fifth fingers similarly affected ; right foot, fourth toe discoloured and patch of skin necrosis on dorsum (Fig. 1). These affected areas were warmer to touch than their appearances suggested. Peripheral pulses normal.

*Treatment.*—During following week discoloration of right hand and of all involved digits became more marked : gangrenous change was considered imminent. Application of heat and the use of vaso-dilators failed to improve affected parts. Penicillin sodium 50,000 units intramuscularly every six hours was commenced. This was followed by a febrile reaction of three days' duration, maximum temperature 103° F. Right hand became very swollen and red line of demarcation clear : amputation considered but postponed owing to poor condition of child : Blood Wasserman reported strongly positive. The penicillin therapy was augmented by daily injections of 0.25 mil. bismostab and potass. iodide by mouth. Following this there was a marked improvement in general health and some improvement in local lesions. Nevertheless dry gangrene ensued, although the affected area was less extensive than originally expected.

On March 22nd, 1948, the child was transferred to Bristol Royal Hospital for Sick Children. On admission, marked rhinitis ; typical facies ; extremities, dry gangrene of right hand, sharp demarcation ; finger tips, third, fourth and fifth digits left hand black, with necrosis of nails : no lesions on right foot : peripheral pulses normal. X- ays of the long bones showed syphilitic osteo-periostitis; Wassermann and Kahn strongly positive ; no haemoglobinuria. A further course of penicillin was begun, 60,000 units every three hours.

One week later, as there was no improvement in the right hand, amputation was performed at proximal metacarpo-phalangeal joint of thumb and through metacarpals of the remainder of the hand. The stump healed well by granulation. The affected fingers of the left hand rapidly regained normal colour and the nails separated. A three-weeks' course of penicillin was followed by sulphostab 0.01 gramme

PLATE XVI

FIG. 1.
Gangrene of right hand in congenital syphilis: note facies.

FIG. 2.

FIG. 3.

DWARFISM (Brailsford-Morquio)

PLATE XVII

Fig. 4.

Fig. 5.

PROGERIA (aged 10)

PLATE XVIII

FIG. 6.

INFANT CRETIN.

PLATE XIX

FIG. 7.
CRETIN, two years old.

FIG. 8.
SAME CRETIN, after treatment.

weekly and bismostab 0.01 bi-weekly: C.S.F. Wasserman negative, Lange colloidal gold curve normal.

The child made good progress and after twelve weeks the blood Wasserman and Kahn reactions were negative, and the rhinitis ceased. On June 28th, 1948, he was discharged from hospital on a course of weekly injections of sulpharsphenamine by his own doctor. Follow-up: six months after discharge the child appeared well and seemed mentally normal for his age. January, 1949. General condition good: further course of penicillin given, followed by sulpharsphenamine. Intelligence good; no residual effects of the disease apart from nasal depression; right-hand stump very good and metacarpal bones normal; skeleton normal by X-ray.

### II: DWARFISM

In 1928 Brailsford in England and Morquio in South America described a peculiar dystrophy characterized by defective development of the skeletal tissues. The infant may appear normal but in childhood deformity is revealed (Figs. 2, 3). Charles and Gerald are identical twins and present the typical posture, and the front view indicates how the spinal shape is adaptable to supporting hands on knees. Movements at joints are variably limited, but a convenient degree of activity is always possible. X-rays show diagnostic irregularities in the epiphyses and especially in the bodies of the vertebrae, osteo-chondro-dystrophy. Intelligence is quite normal but dwarfism results.

### III: PROGERIA

Progeria, (Hastings Gilford, 1904) is always recognizable on sight. All children so affected look very similar (Figs. 4, 5). The name is self-descriptive and the illustrations of our case leave no doubt about the premature ageing. The tissue changes, including bones, muscles and viscera, are all similar to those found in senility, and likewise the pituitary gland shares the atrophy. Curiously enough, the appetite is well preserved and may even be excessive, despite the progressive loss in weight. The underlying endocrine and metabolic factors are complex and little understood. A very significant effect of the disease is arterio-sclerosis: the coronary arteries may become calcified and the child at 12 years may succumb to coronary thrombosis (as happened in this case).

BERYL D. CORNER, M.D., M.R.C.P.
*Paediatrician, United Bristol Hospitals and Southmead Hospital*

---

### IV: CRETINISM

**Case 1.**—CRETINISM IN AN INFANT AGED 3 MONTHS. P.H., aged 3 months, weight 10 lb., brought to Children's Hospital for failure to thrive and constipation : showed typical features of cretinism. Fig. 6 shows myxoedematous face, with swollen eyelids and generalized thickening of subcutaneous tissue ; there is a large umbilical hernia. The child's father was diagnosed as a cretin by Dr. Carey Coombs at age 6 months and has been on thyroid therapy since.

**Case 2.**—CRETINISM IN CHILD AGED 2 YEARS. J.S., aged 2 years, noticed by her parents to be late in walking and talking. Fig. 7 shows typical features of cretinism : straight dry hair, thickened eyelids, cheeks and lips, thick protruding tongue, prominent abdomen with umbilical hernia, thickened skin of legs and arms. Fig. 8, from a photograph taken six weeks after thyroid therapy, shows improved condition of hair, loss of thickened subcutaneous tissues of face and limbs, less prominence of abdomen and diminution in size of umbilical hernia.

### V : MONGOLISM

MONGOLISM. M.D., girl aged 1 year ; second child in family : parents both aged 40. The illustrations show features of mongolism : brachycephalic skull, depressed bridge of nose, slanting orbits, widely open mouth, short fingers with incurved terminal phalanx of fifth finger, Figs. 9, 10, 11. (I am indebted to Professor Neale for permission to use these photographs.)

JOHN LOWE, M.D., F.R.C.S.
*Medical Superintendent, St. Margaret's Hospital,*
*Stratton St. Margaret, Wilts.*

### VI: GARGOYLISM

(Hurler's Syndrome ; Lipochondrodystrophy ; Dysostosis multiplex)

IN September, 1938, I was asked to see twin girls, aged 4 years, who were stated to present clinical appearances of congenital syphilis, but in whom Wassermann reaction was negative. Both parents were healthy—husband aged 31 years, wife aged 29 years ; there was no history of abnormality in either family tree, and no consanguinity. There were four children ; a normal boy aged 7, Richard aged 5½, and twin-girls aged 4. Richard and the twins were obviously victims of a curious disease ; the contrast between the healthy child and those affected is well shown in Figs. 12, 13.

*Clinical Description.*—The outstanding characteristics of the condition in the affected children were dwarfism, angular kyphosis in the dorso-lumbar region and an attitude of flexion of joints, particularly noticeable in elbows, knees and fingers : head abnormally

FIG. 9.

FIG. 10.

FIG. 11.

MONGOLISM.

PLATE XXI

Fig. 12.

GARGOYLISM. Three children with normal brother.

PLATE XXII

Fig. 13.

GARGOYLISM. Dwarf; large head, short neck, gross features angular kyphosis, flexion of limb joints; protuberant abdomen umbilical hernia; open mouth, large tongue, "miserable."

large, neck short and thick, features gross and ugly. The children looked unhappy, were given to crying and were obviously subnormal mentally. Speech was thick and guttural; scalp hair was fine and silky; the ears large and situated low on head. The eyes were widely separated and prominent, and the corneæ had a curious misty appearance; supraorbital ridges well marked and superabundance of hair on eyebrows. The nose was broad, short and had a sunken bridge; nostrils unduly open, with profuse seropurulent discharge: long upper lip, mouth open, tongue large, teeth irregular and widely spaced, palate highly arched. In all three children the abdomen was protuberant, liver and spleen greatly enlarged and umbilical hernia was present.

Richard was admitted to hospital for further investigation. W.R. was negative. Height was 32 inches; from vertex to umbilicus measured 18 inches, from umbilicus to heel 14 inches; weight was 29 lb. Skull: circumference (above ears) 20½ inches, bitemporal measurement 12 inches.

X-ray findings.—Skull: a normal pituitary fossa, nasal bones depressed, antra undeveloped, ethmoids opaque. Spine: marked deformities of the bodies of the upper lumbar vertebrae and sacrum, especially kyphosis; ribs flatter than normal. Femora: sclerosis of upper third and coxa valga; sclerosis of lower left metaphysis. Left tibia: sclerosis of upper metaphysis. Hands: marked deformity of metacarpals and phalanges; the bones were widened and irregular and cystic changes were seen. Feet showed similar changes, but less marked.

Richard had several attacks of severe abdominal pain, thought to be due to his umbilical hernia, and as this had become incarcerated, an operation was carried out by Mr. J. E. Schofield on January 19th, 1939. He confirmed the enlargement of liver and spleen and also reported a large number of glands, one of which was removed for section; report stated "many mononuclear cells present". Patient was discharged from hospital on March 29th; no recurrence of his abdominal symptoms.

So far as is known no treatment is of any use. All three children died from broncho-pneumonia, Richard when aged 7, one girl aged 5½ and the other twin aged 5 years. Two other children have since been born, one boy is aged 5½ years and another boy who is now 8 months old. So far no obvious defects have been found in these two children, with the exception of an easily palpable liver.

*Commentary.*—This curious condition is often referred to as Hurler's syndrome, although it was first described by Hunter in 1917. Ellis, Sheldon and Capon in 1936 were so impressed by the resemblance to gargoyles that the condition is now known as Gargoylism. The condition is familial, but there is no consensus of opinion as to the actual cause. Some authorities assert that the disturbance in endochondral growth is due to a defect in the germ plasm, while others regard the condition as being primarily due to disease of lipoid metabolism, and classify the disease along with Niemann-Pick, Tay-Sachs, Hand-Schuller-Christian, and Gaucher's disease. More recently Strauss and others have drawn attention to the hypertrophy of fascia, and they state that "it seems that disease of collagen chiefly of fascia and ligaments is a more adequate explanation for the physical deformities than any previously considered. . . . Lipoidosis is not a constant feature of the disease entity. When present, however, it is found in the brain and in the reticulo-endothelial system as in other idiopathic lipoid dystrophies. Chemical analysis of the tissues of our case revealed a significant increase in lipid content of the lymph nodes, but not of the brain, liver or spleen. The increased lipid was, by exclusion, simple fat, probably in complex protein combination. The fact that it was not a phospholipid, cerebroside, or cholesterol, separates this disturbance from the other idiopathic lipoid dystrophies. No relationship was established between the genesis of the physical abnormality and the reticulo-endothelial disease ".

*Diagnosis.*—The general appearance may suggest congenital syphilis, but the kyphosis and flexion attitude are not seen with that ; and the Wassermann reaction is negative. Cretinism may be suggested by adiposity and mental deficiency, but thyroid administration makes no improvement. Greene and Rundle contrast the two conditions thus : " the gargoyle's face may resemble the cretin's rather strongly and umbilical hernia may be present, but the other characteristics of cretinism are absent ; in particular the skin is normal. In the cretin abnormal soft parts are moulded on a normal skeleton, whereas in the gargoyle normal soft parts cover the abnormal skeleton ". Rickets may be simulated by large head, protuberant abdomen, genu valgum, etc., but in gargoylism there is *restriction* of movement of the joints rather than increased laxity, and calcium and phosphorus content of blood is normal. Morquio and Brailsford have described a condition of chondrodysplasia which may resemble gargoylism (see above II) : but in which there is no mental deficiency, liver and spleen are not enlarged and corneæ are unaffected, while there is no swelling round the joints in gargoylism.

### REFERENCES

Ellis, R. W. B., Sheldon, W., and Capon, N. B. (1936), *Quart. J. Med.*, **5**, 119.

Greene, R., and Rundle, F. F. (1948), *Pract. of Endocrinology, London.* 248.

Hunter, C. (1917), *Proc. Roy. Soc. Med.* (*Section for Study of Disease in Children*), **10** ; 104–116.

Hurler, G. (1920), *Z. Kinderheilk*, **24**, 220.

Strauss, R., Merliss, R., and Reiser, R. (1947), *American Journal Clin. Path.*, **9** ; 671–694. (Extensive bibliography.)

## Figures and Tables

**Fig. 1. f1:**
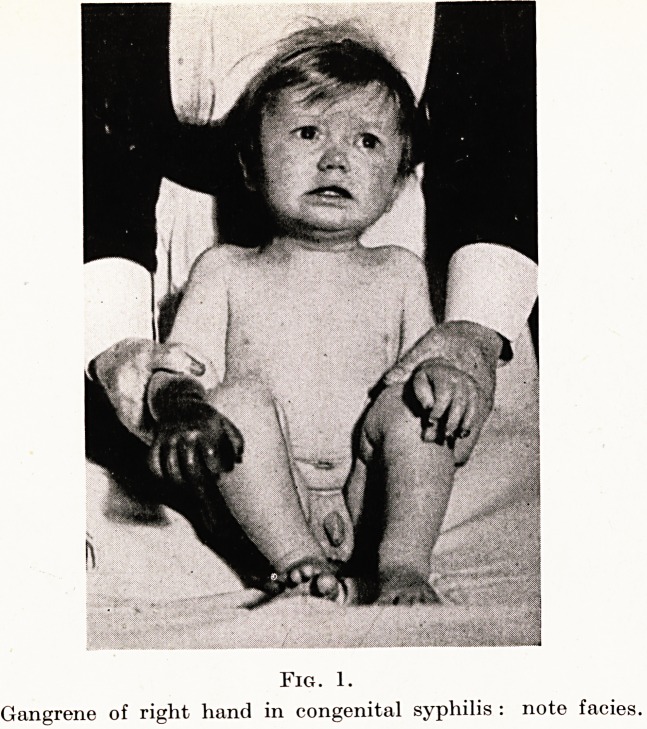


**Fig. 2. Fig. 3. f2:**
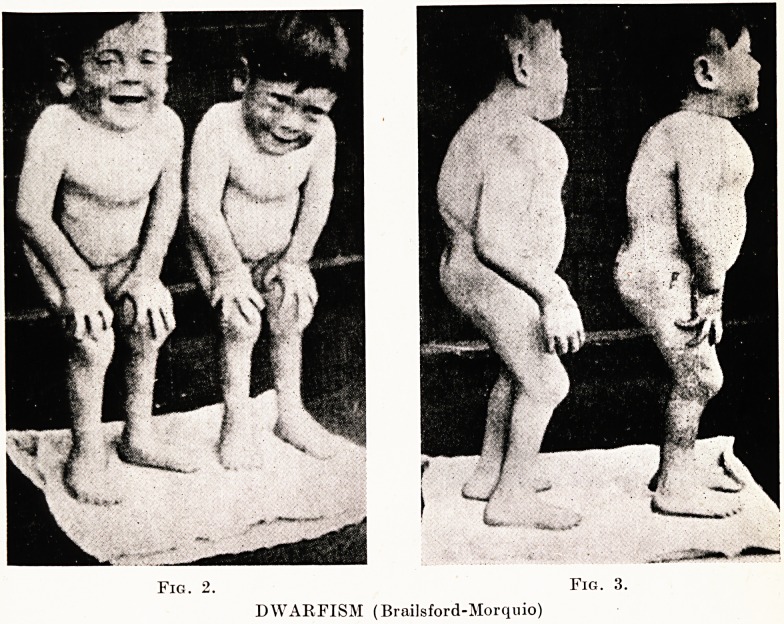


**Fig. 4. Fig. 5. f3:**
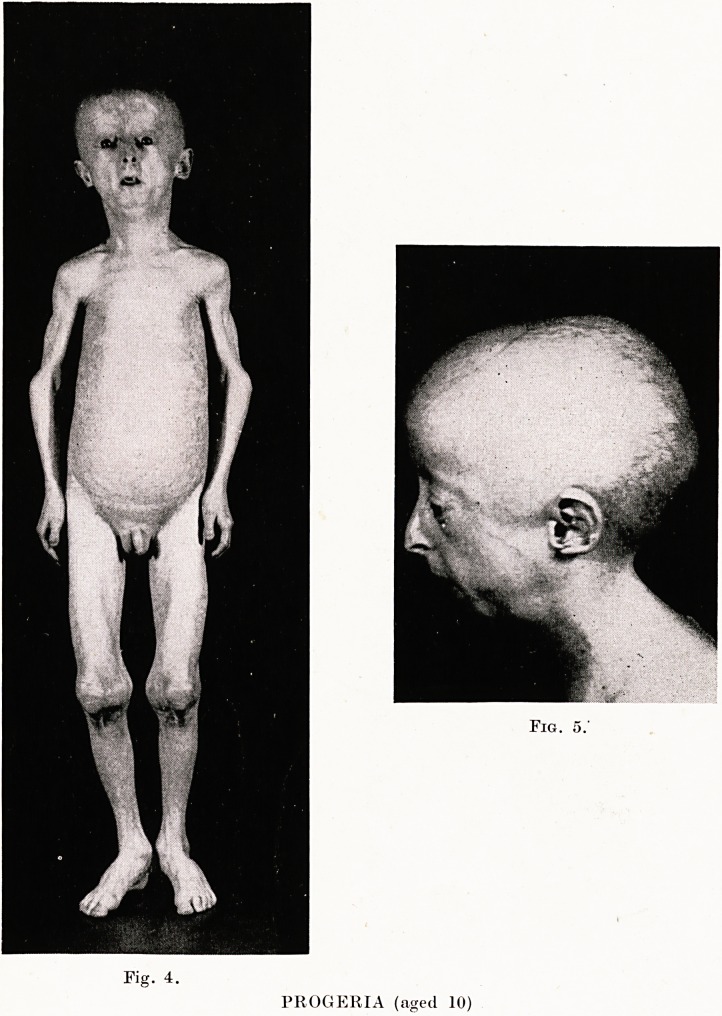


**Fig. 6. f4:**
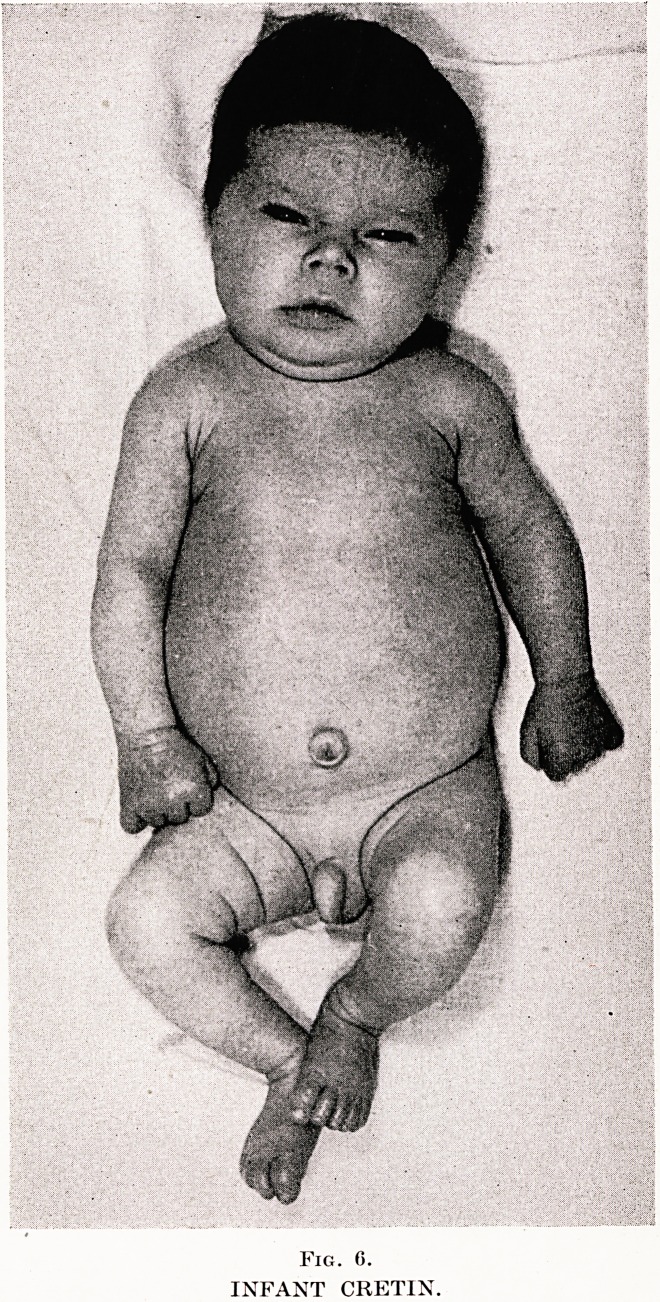


**Fig. 7. f5:**
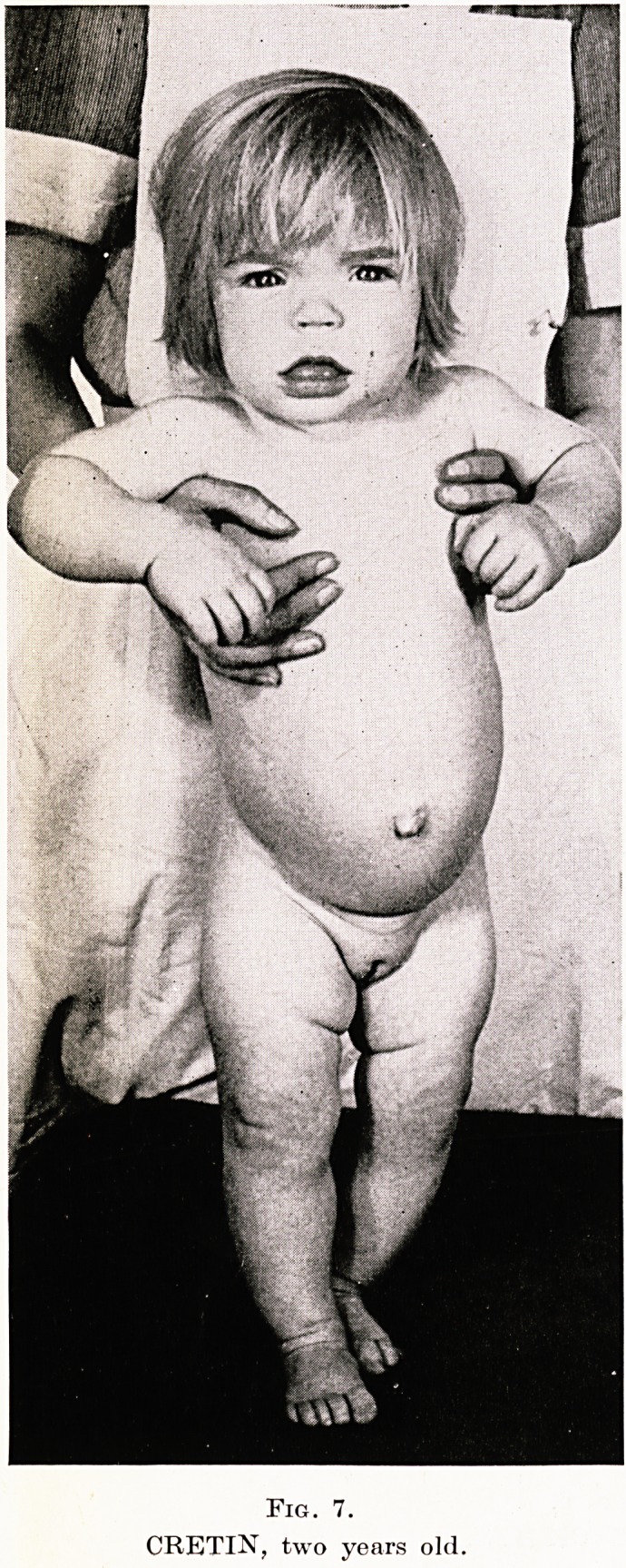


**Fig. 8. f6:**
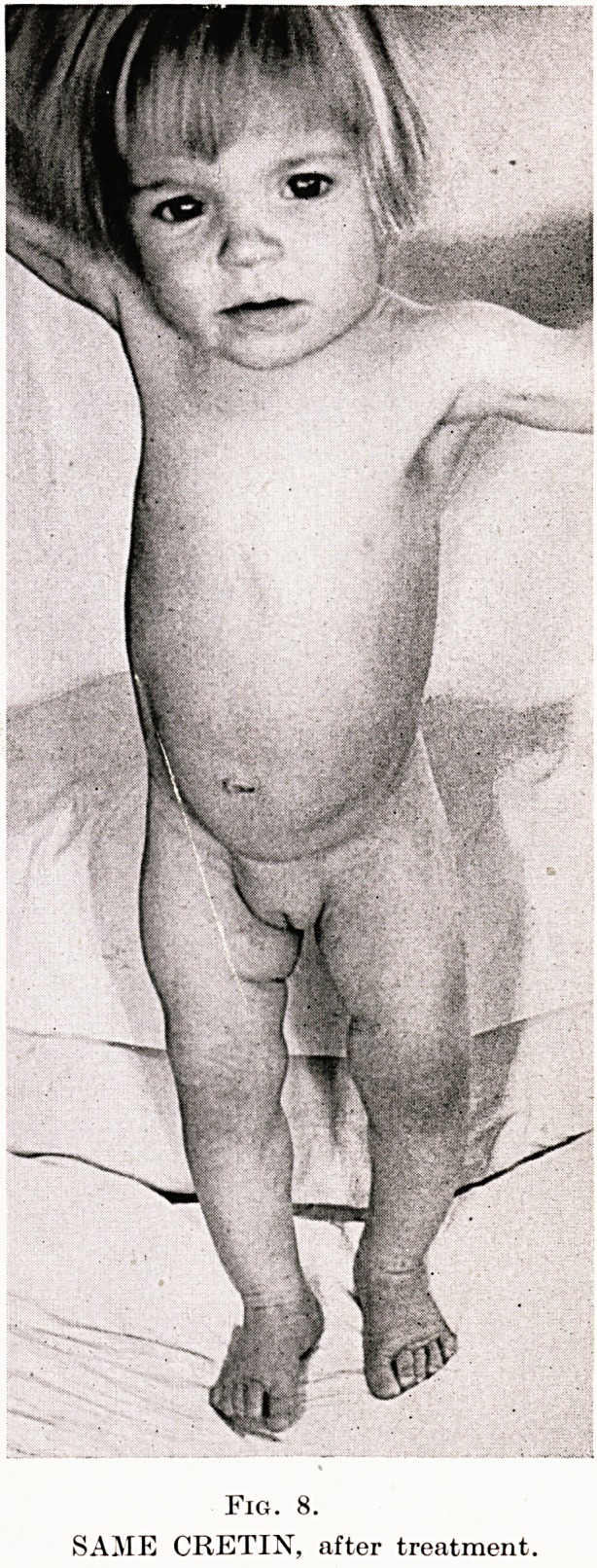


**Fig. 9. Fig. 10. Fig. 11. f7:**
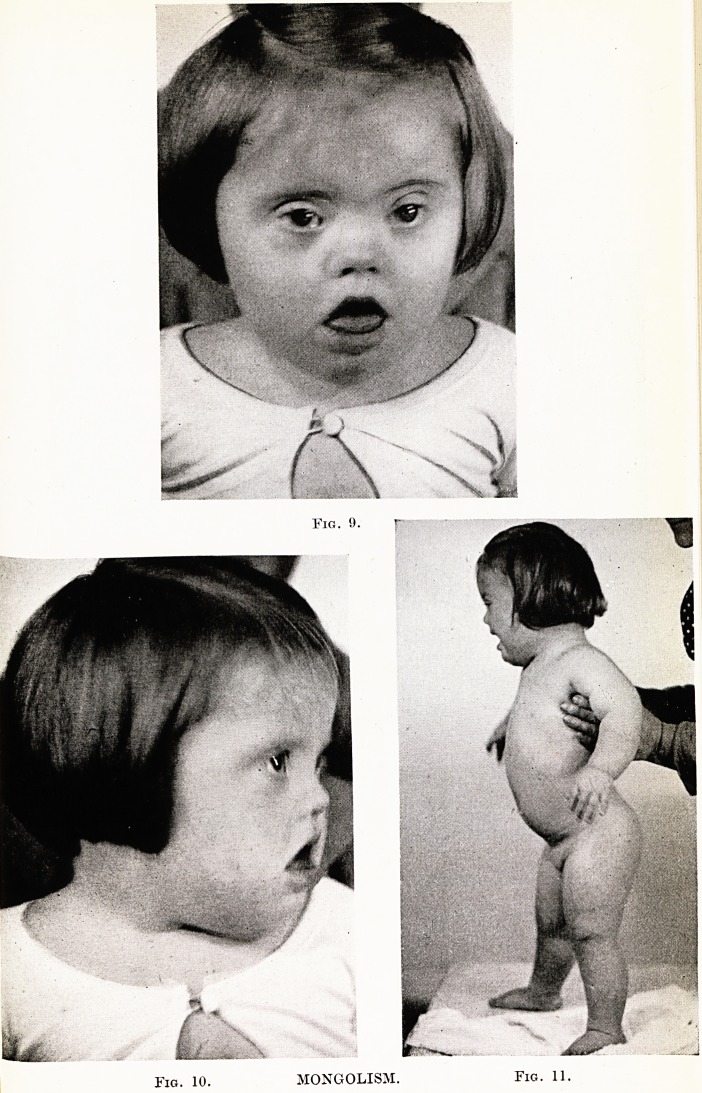


**Fig. 12. f8:**
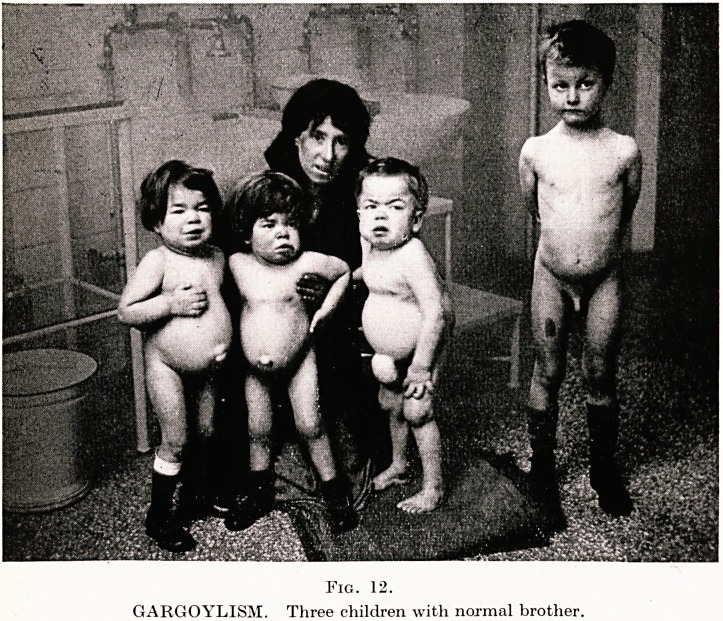


**Fig. 13. f9:**